# The role of lifestyle and non-modifiable risk factors in the development of metabolic disturbances from childhood to adolescence

**DOI:** 10.1038/s41366-020-00671-8

**Published:** 2020-09-17

**Authors:** Claudia Börnhorst, Paola Russo, Toomas Veidebaum, Michael Tornaritis, Dénes Molnár, Lauren Lissner, Staffan Mårild, Stefaan De Henauw, Luis A. Moreno, Anna Floegel, Wolfgang Ahrens, Maike Wolters

**Affiliations:** 1 Leibniz Institute for Prevention Research and Epidemiology-BIPS, 28359 Bremen, Germany; 2 Institute of Food Sciences, National Research Council, 83100 Avellino, Italy; 3 National Institute for Health Development, Estonian Centre of Behavioral and Health Sciences, 11619 Tallinn, Estonia; 4 Research and Education Institute of Child Health, 2035 Strovolos, Cyprus; 5 Department of Pediatrics, Medical School, University of Pécs, 7623 Pécs, Hungary; 6 Section for Epidemiology and Social Medicine, Department of Public Health and Community Medicine, Institute of Medicine, Sahlgrenska Academy, University of Gothenburg, 40530 Gothenburg, Sweden; 7 Department of Paediatrics, Institute of Clinical Sciences, Sahlgrenska Academy at Gothenburg University, 41685 Gothenburg, Sweden; 8 Department of Public Health, Ghent University, 9000 Ghent, Belgium; 9 GENUD (Growth, Exercise, Nutrition and Development) Research Group, Faculty of Health Sciences, Universidad de Zaragoza, Instituto Agroalimentario de Aragón (IA2), Instituto de Investigación Sanitaria Aragón (IIS Aragón), Centro de Investigación Biomédica en Red de Fisiopatología de la Obesidad y Nutrición (CIBERObn), 50009 Zaragoza, Spain; 10 Institute of Statistics, Faculty of Mathematics and Computer Science, University of Bremen, 28359 Bremen, Germany

**Keywords:** Obesity, Risk factors

## Abstract

**Background:**

The study aimed to identify the effects of lifestyle, C-reactive protein (CRP) and non-modifiable risk factors on metabolic disturbances in the transition from childhood to adolescence.

**Methods:**

In 3889 children of the IDEFICS/I.Family cohort, latent transition analysis was applied to estimate probabilities of metabolic disturbances based on waist circumference, blood pressure, blood glucose, and lipids assessed at baseline and at 2- and 6-year follow-ups. Multivariate mixed-effects models were used to assess the age-dependent associations of lifestyle, non-modifiable risk factors and CRP, with the transformed probabilities of showing abdominal obesity, hypertension, dyslipidemia, or several metabolic disturbances (reference: being metabolically healthy).

**Results:**

Higher maternal body mass index, familial hypertension as well as higher CRP *z*-score increased the risk for all four metabolic outcomes while low/medium parental education increased the risk of abdominal obesity and of showing several metabolic disturbances. Out of the lifestyle factors, the number of media in the bedroom, membership in a sports club, and well-being were associated with some of the outcomes. For instance, having at least one media in the bedroom increased the risk for showing several metabolic disturbances where the odds ratio (OR) markedly increased with age (1.30 [95% confidence interval 1.18; 1.43] at age 8; 1.18 [1.14; 1.23] for interaction with age; i.e., resulting in an OR of 1.30 × 1.18 = 1.53 at age 9 and so forth). Further, entering puberty at an early age was strongly associated with the risk of abdominal obesity (2.43 [1.60; 3.69] at age 8; 0.75 [0.69; 0.81] for interaction with age) and the risk of showing several metabolic disturbances (2.46 [1.53; 3.96] at age 8; 0.71 [0.65; 0.77] for interaction with age).

**Conclusions:**

Various factors influence the metabolic risk of children revealing the need for multifactorial interventions. Specifically, removing media from children’s bedroom as well as membership in a sports club seem to be promising targets for prevention.

## Introduction

As a consequence of the worldwide obesity epidemic, metabolic disturbances such as dyslipidemia, hypertension, and insulin resistance are on the rise already in childhood and adolescence and the prevalence of metabolic syndrome (MetS) in children is also increasing [1]. Lifestyle factors such as a high consumption of processed and snack foods and a low consumption of vegetables and wholemeal products [2, 3], lack of physical activity (PA), high levels of media use and sedentary behaviors (SB) [4, 5] as well as low well-being e.g., due to psychosocial stress [6, 7] are associated with an increased risk for overweight, obesity and metabolic disturbances. Apart from lifestyle, non-modifiable^1^ risk factors such as family history of MetS components, parental education, maternal body mass index (BMI), breastfeeding duration, birth weight as well as pubertal development also influence the risk of metabolic disturbances [8–12]. Additionally, elevated serum C-reactive protein (CRP), which is probably a mediator between lifestyle factors and certain health outcomes, contributes to the pathogenesis of metabolic disturbances, e.g., by increasing the risk for hypertension, insulin resistance, and for a high proportion of abdominal fat [13–16].

Further, several studies have indicated that metabolic disturbances often persist from childhood to adolescence and adulthood [17–20]. This results in increased morbidity and cardiovascular risk, whereas the remission of temporal occurrence of metabolic disturbances seems to normalize the risk [21]. Besides the adverse impact of obesity, the persistence of metabolic disturbances may be enhanced by continuing adherence to unfavorable behaviors such as unhealthy dietary patterns [22] and low PA [23, 24], which often manifest during childhood and also track into adolescence/adulthood.

To account for the age trend of metabolic parameters during childhood, a new definition of MetS and of disturbances in its single components was proposed for children by Ahrens et al. [1]. Using the corresponding age- and sex-specific cut-offs, we investigated metabolic statuses in children and during their transition to adolescence using latent transition models in a previous paper [25]. The present paper builds on the previous one by studying the effects of lifestyle factors such as dietary behavior, PA, media use and well-being, and of non-modifiable factors^2^such as parental education and certain early life factors as well as of serum CRP on metabolic risk statuses during childhood and adolescence.

## Methods

### Study population and data

The IDEFICS (Identification and Prevention of Dietary- and Lifestyle-Induced Health Effects in Children and Infants)/I.Family cohort is a multicenter population-based study aiming to investigate the causes of diet- and lifestyle-related diseases in children, adolescents, and their families. The baseline survey (T0) was conducted in 2007/2008 in eight European countries (Belgium, Cyprus, Estonia, Germany, Hungary, Italy, Spain, and Sweden) and included children aged 2–9 years. In total, 16,229 children fulfilling the inclusion criteria participated. The survey included interviews with parents concerning lifestyle habits and dietary intakes of their children, physical examinations of the children as well as the collection of blood samples. All measurements and samples were taken using standardized procedures in all eight countries. Details on the design and objectives of the IDEFICS/I.Family study can be obtained from Ahrens et al. [26, 27]. A follow-up examination (T1) was conducted in 2009/2010 and the same standardized assessments were applied in 13,596 children, 2555 newcomers and 11,041 who had participated at T0. A second follow-up examination (T3^3^) took place in 2013/2014, where 7105 of the children participating already in T0 or T1 were included [27]. Before children entered the study, parents provided written informed consent. Additionally, children aged 12 years and older gave written consent, while younger children gave oral consent for the examinations and sample collection. Ethical approval was obtained from the institutional review boards of all eight study centers.

### Outcome measures: MetS components

Details on anthropometric measurements, blood pressure measurements, and collection of blood markers are given in Supplementary Material S1.

According to previously described methods [28–30], age- and sex- (for blood pressure also height-) specific reference values were derived for diastolic (DBP) and systolic blood pressure (SBP), waist circumference, high-density lipoprotein (HDL) cholesterol, triglycerides, and blood glucose in children and adolescents, using the data collected in the IDEFICS/I.Family cohort. As the laboratory methods to measure blood glucose, HDL and triglycerides changed between T0/T1 and T3 (see Supplementary Material S1), separate reference curves were estimated for T0/T1 and T3, respectively, and used for the analysis. Subsequently, children were defined as being above the so-called “monitoring” or “action” levels of the different metabolic parameters, if their parameters exceeded the 90th or 95th age- and sex-specific reference percentiles (age-, sex-, and height specific in case of blood pressure), respectively [1]. In the present investigation, waist circumference was considered as a marker for abdominal obesity, SBP and DBP as markers for hypertension (criterion: either SBP or DBP above 90th/95th percentile for monitoring/action level), triglycerides and HDL cholesterol for dyslipidemia (criterion: either triglycerides above 90th/95th percentile or HDL below 10th/5th percentile) and fasting blood glucose as a marker for disturbances of the glucose metabolism.

### Determinants of MetS

Various potential determinants of MetS that are described in detail in Supplementary Material S2 were selected a priori according to previous literature.

We considered the following as lifestyle factors: consumption frequencies of fruits and vegetables (times/day), consumption frequencies of processed foods (times/day), a psychosocial well-being score (range: 0–48; a higher score indicating a higher well-being), membership in a sports club (yes vs no), and the number of media devices in the child’s bedroom (modeled as 0 vs ≥1). We selected the last two as proxy variables for PA and SB, as objectively measured data were only available in a small proportion of children included in this analysis. Membership in a sports club has been shown to be associated with objectively measured time spent in moderate-to-vigorous PA [31–33], and the presence of media devices in the personal space has been shown to increase the risk of higher screen time in children [34].

In addition, the following non-modifiable risk factors were considered: age of the child, sex, country of residence, highest educational level of parents according to the International Standard Classification of Education (ISCED; modeled as dummy, i.e., low/medium vs high) [35], maternal BMI, family history of diseases (hypertension, dyslipidemia and type 2 diabetes; yes vs no), birth weight (g), total breastfeeding duration (in months), and pubertal status (yes vs no).

### C-reactive protein (CRP)

For the assessment of serum high-sensitive CRP concentrations, latex-enhanced nephelometry (BN2-Nephelometer, Siemens, Eschborn, Germany) was used at T0 and T1. The serum CRP values were measured with a precision of 0.1 mg/l and a lower detection limit of 0.2 mg/l. At T3, CRP levels were determined by enzyme-linked immunosorbent assay, using electrochemiluminescent multiplex assays (Meso Scale Discovery, Rockville, USA) and a lower detection limit of 0.0007 mg/l. CRP, which is used as a marker for inflammation, was transformed to an age- and sex-specific *z*-score according to previously described methods [36] and separate reference curves depending on the laboratory method used were applied.

### Analysis dataset

Laboratory measurements obtained from non-fasting blood samples were not considered (1897 measurements from 1408 children). None of the children in the dataset had acute infections/inflammation, defined as a CRP level ≥10 mg/l. In addition, children taking medications that might have influenced the parameters of interest for this study were excluded. For the latter purpose, children being treated for type1/type 2 diabetes (ATC codes: A10A, A10B, A10X), elevated blood lipids (C10), hypertension (C02, C03, C07, C08, C09), or obesity (A08) were identified based on ATC codes and excluded (*N* = 54 subjects).

Our analysis dataset included 3889 children aged ≥4 to ≤15 years across all examination waves, who participated at baseline and T3 and who provided at least two measurements of all MetS components.

### Statistical methods

As described in detail elsewhere [25], latent transition analysis (LTA [37]) was used to identify groups of children with distinct metabolic statuses at the three examination points. LTA is a longitudinal extension of latent class analysis that enables the estimation of transition probabilities among latent statuses (distinct metabolic statuses in this case) over time [37]. The probabilities of being assigned to the different latent metabolic statuses at T0, T1, and T3 were estimated using the variables reflecting the classification of the children for the four MetS components (normal level, above monitoring or above action level) as already described. Models with 3 up to 7 latent statuses were estimated, with the 5-status model showing the best fit (evaluated based on the Bayesian Information Criterion). The five latent statuses were labeled and characterized as follows (see also [25]):

1. Metabolically healthy: high probability of all markers being within the normal range
2. Abdominal obesity: low probability of having normal levels for waist circumference but high for the other metabolic markers
3. Dyslipidemia: low probability of having normal lipid levels but high probabilities of having normal levels for the other three markers
4. Hypertension: low probability of showing normal blood pressure values but high probabilities of having normal levels for the other three markers
5. Several MetS components: low probability of having normal waist circumference and rather low probabilities of normal levels for the other metabolic markers

Let p_healthy_ denote the probability of children being assigned to the metabolically healthy status and analogously p_abdominal_obesity_, p_dyslipidemia_, p_hypertension_, and p_MetS_ the probabilities for the other statuses_._ As these probabilities sum up to 1, our data can be considered as compositional data and were analyzed as such following the approach described in Faes et al. [38]. Children’s probabilities of being assigned to the different latent statuses at T0, T1, and T3 were transformed using the additive logratio transformation:

- p_abdominal_obesity_ is transformed to ln(p_abdominal_obesity_/p_healthy_)
- p_dyslipidemia_ is transformed to ln(p_dyslipidemia_/p_healthy_)
- p_hypertension_ is transformed to ln(p_hypertension_/p_healthy_)
- p_MetS_ is transformed to ln(p_MetS_/p_healthy_)

The transformed values were then used as outcome variables in a multivariate analysis. This enables the interpretation of results after backtransformation in terms of odds ratios (OR), with the metabolically healthy status serving as the reference group for all other statuses. Multivariate mixed-effects models were used to assess the age-dependent associations between lifestyle factors, non-modifiable risk factors and CRP and the transformed probabilities of having abdominal obesity, hypertension, dyslipidemia, or several MetS components, respectively, considering T0, T1, and T3 simultaneously (SAS, Proc MIXED). Multiple imputation was applied for missing covariates (see Supplementary Material S3/S4). The model included a random subject-specific intercept and accounted for the repeated measurements, where a variable indicating the examination wave was added to the “repeated” statement (to allow for the correlation of measurements taken from the same child). The factor-analytic covariance structure was chosen for the random effects to allow modeling heterogeneous covariances (when using the preferred unstructured covariance structure, the estimated G matrix was not positive definite). Continuous co-variables were centered before model fit to obtain meaningful model estimates.

A first model including the following factors was run: all lifestyle factors (fruit/vegetable consumption centered to 5 times/day, processed food consumption centered to 0 times/day, being a member of a sports club (ref: yes), well-being score centered to 40, having at least 1 media in bedroom (ref: 0 media)) as well as all non-modifiable risk factors (birth weight centered to 3500 g, breastfeeding centered to 6 months, maternal BMI centered to 23 kg/m^2^ [2], low/medium parental ISCED level (ref: high), country of origin, age centered to 8 years, female sex (ref: male), entered puberty (ref: no), family history of diabetes, hypertension and dyslipidemia (ref: no for each)), and the interaction terms of these variables with age. Age interactions with *p* ≥ 0.10 for all of the four outcomes were removed from the model in a second step (applied only to the age interaction for processed food consumption). As CRP was assumed to lie on the causal pathway between lifestyle factors and the four health outcomes, CRP *z*-score (centered to 0) was added to the above model in a subsequent step [39], again including an age interaction. As overweight and obesity can also lead to increased CRP, we performed some sensitivity analyses for the outcomes dyslipidemia and hypertension with additional adjustments for BMI *z*-score (Supplementary Material S5).

All analyses were performed using SAS^®^ statistical software version 9.3 (SAS Institute, Inc., Cary, NC, USA). Proc LTA was used to conduct the LTA, Proc MI for the multiple imputation and Proc MIXED for the multivariate mixed-effects models. Estimates for the multiple imputed datasets were combined using Proc MIANALYZE.

## Results

The proportions of children assigned to the different latent metabolic statuses at T0, T1, and T3 are shown in Table 1. The majority of children was classified as metabolically healthy at all time points (67.8% at T0, 61.7% at T1, and 59.8% at T3); the proportions of children assigned to the dyslipidemia and hypertension groups were small (all below 7%). At T0, T1, and T3, the prevalence of abdominal obesity was 15.2%, 17.4%, and 17.5%, respectively. Further, the prevalence of showing several MetS components was markedly higher at T1 (9.1%) and T3 (10.6%) compared to at T0 (5.6%).

**Table 1:** Prevalence of the different latent metabolic statuses at T0, T1, and T3 for the total study group and stratified by sex.

		All		Male		Female	
	Wave	*N*	%	*N*	%	*N*	%
Metabolically healthy	T0	2635	67.8	1317	66.6	1318	69.0
	T1	2399	61.7	1203	60.8	1196	62.6
	T3	2525	64.9	1261	63.7	1264	66.2
Abdominal obesity	T0	589	15.2	305	15.4	284	14.9
	T1	675	17.4	339	17.1	336	17.6
	T3	682	17.5	344	17.4	338	17.7
Dyslipidemia	T0	257	6.6	135	6.8	122	6.4
	T1	244	6.3	131	6.6	113	5.9
	T3	166	4.3	84	4.2	82	4.3
Hypertension	T0	192	4.9	101	5.1	91	4.8
	T1	216	5.6	108	5.5	108	5.7
	T3	105	2.7	51	2.6	54	2.8
Several MetS components	T0	216	5.6	121	6.1	95	5.0
	T1	355	9.1	198	10.0	157	8.2
	T3	411	10.6	239	12.1	172	9.0

*MetS* metabolic syndrome.

A description of the covariates stratified by the five metabolic statuses is given in Table 2. The metabolically healthy group generally showed the most favorable mean levels of covariates, although the differences were small.

**Table 2:** Description of the study population: means (SD) for continuous variables and numbers and percentages for categorical variables in the different latent metabolic groups (imputed dataset).

Status at T0	All (*N* = 3889)		Metabolically healthy (*N* = 2635)		Abdominal obesity (*N* = 589)		Dyslipidemia (*N* = 257)		Hypertension (*N* = 192)		Several MetS components (*N* = 216)	
Covariate at T0	Mean	SD	Mean	SD	Mean	SD	Mean	SD	Mean	SD	Mean	SD
Maternal BMI	23.9	4.3	23.4	4	25.4	4.9	24	4.5	23.8	4	26.2	4.8
Max ISCED of both parents	2.5	0.6	2.5	0.6	2.3	0.7	2.5	0.6	2.5	0.6	2.2	0.6
Breastfeeding [months]	5.7	6.6	5.9	6.5	4.9	6.6	5.9	7	7.3	7.5	4.1	5.5
Birth weight (g)	3345.8	566.1	3341.8	559.7	3371.6	590.4	3341.5	558.2	3343.7	574.3	3331.3	580.6
Fruit/veg [times per day]	2.7	1.7	2.7	1.7	2.6	1.7	2.9	2	2.7	2.1	2.6	1.7
Preserved food [times per day]	1	0.8	1	0.8	1	0.8	1	0.7	1.2	1	1.1	1
Number of media in bedroom	0.9	1.2	0.8	1.2	1.2	1.3	0.9	1.3	0.9	1.3	1.2	1.4
Well-being score	39.8	4.7	40.1	4.5	39.1	4.9	40	4.9	39.2	4.7	38.8	4.8
CRP *z*-score	0.4	0.8	0.3	0.8	0.7	0.9	0.4	0.9	0.2	0.8	1	0.9
Covariate at T0	*N*	%	*N*	%	*N*	%	*N*	%	*N*	%	*N*	%
Member in sports club	1954	50.2	1339	50.8	293	49.8	117	45.5	96	50	109	50.5
Not member in sports club	1935	49.8	1296	49.2	296	50.3	140	54.5	96	50	107	49.5
No familial hypertension	3096	79.6	2163	82.1	443	75.2	209	81.3	139	72.4	142	65.7
Familial hypertension	793	20.4	472	17.9	146	24.8	48	18.7	53	27.6	74	34.3
No familial diabetes	3694	95	2520	95.6	558	94.7	238	92.6	179	93.2	199	92.1
Familial diabetes	195	5	115	4.4	31	5.3	19	7.4	13	6.8	17	7.9
No familial dyslipidemia	3288	84.6	2261	85.8	494	83.9	207	80.5	157	81.8	169	78.2
Familial dyslipidemia	601	15.5	374	14.2	95	16.1	50	19.5	35	18.2	47	21.8

*CRP* C-reactive protein, *ISCED* International Standard Classification of Education, *MetS* metabolic syndrome.

The ORs for the (mutually adjusted) associations between our exposures and the metabolic outcomes are presented in Table 3. The non-modifiable risk factors mainly affecting our metabolic outcomes were parental ISCED level, maternal BMI, pubertal status, and family history of hypertension. For instance, children with low/medium parental ISCED level had a 1.14 and 1.25 times higher risk of being in the abdominal obesity or several MetS components groups, respectively, at age 8. A higher maternal BMI increased the risk of showing abdominal obesity (1.29 [1.25; 1.34]; OR [95% confidence interval]), dyslipidemia (1.09 [1.07; 1.11]), hypertension (1.10 [1.07; 1.12]), and several MetS components (1.47 [1.39; 1.55]). Entering puberty at an early age was associated with a higher risk of showing abdominal obesity (2.43 [1.60; 3.69] at age 8; 0.75 [0.69; 0.81] for interaction with age, i.e., resulting in an OR of 0.75 × 2.43 = 1.82 at age 9, 0.75^2^ × 2.43 = 1.37 at age 10 and so forth) as well as a higher risk of showing several MetS components (2.46 [1.53; 3.96]) at age 8; 0.71 [0.65; 0.77] for interaction with age). Strong associations were also found for CRP, where a 1 SD increase in CRP *z*-score increased the risk for all metabolic outcomes at age 8.

**Table 3:** Odds ratios and 95% confidence intervals for the associations of lifestyle factors and C-reactive protein with the risk of showing the four distinct metabolic statuses (reference status: being metabolically healthy); back transformed results of the multivariate mixed-effects models.

	Abdominal obesity (ref: healthy)			Dyslipidemia (ref: healthy)			Hypertension (ref: healthy)			Several MetS components (ref: healthy)		
	OR	LCL	UCL	OR	LCL	UCL	OR	LCL	UCL	OR	LCL	UCL
Age	1.01	0.94	1.09	0.99	0.94	1.04	**1.07**	**1.01**	**1.13**	0.97	0.88	1.08
Entered puberty (yes; ref: no)	**2.43**	**1.60**	**3.69**	**1.62**	**1.09**	**2.42**	1.15	0.76	1.73	**2.46**	**1.53**	**3.96**
Entered puberty × age	**0.75**	**0.69**	**0.81**	**0.86**	**0.80**	**0.93**	**0.91**	**0.84**	**0.98**	**0.71**	**0.65**	**0.77**
ISCED of parents (low medium; ref: high)	**1.14**	**1.00**	**1.29**	1.01	0.91	1.12	1.12	0.99	1.26	**1.25**	**1.05**	**1.49**
ISCED of parents × age	1.01	0.99	1.03	1.03	1.00	1.05	1.01	0.99	1.04	0.99	0.97	1.02
Maternal BMI	**1.29**	**1.25**	**1.34**	**1.09**	**1.07**	**1.11**	**1.10**	**1.07**	**1.12**	**1.47**	**1.39**	**1.55**
Maternal BMI × age	1.00	0.99	1.00	1.01	1.00	1.01	1.00	0.99	1.00	1.00	0.99	1.00
Birth weight	**1.03**	**1.01**	**1.06**	1.00	0.98	1.01	0.99	0.97	1.01	1.03	0.99	1.07
Birth weight × age	1.00	1.00	1.00	1.00	1.00	1.00	1.00	1.00	1.00	1.00	0.99	1.00
Breastfeeding	0.99	0.97	1.02	1.00	0.99	1.01	1.00	0.98	1.01	0.99	0.95	1.03
Breastfeeding × age	1.00	1.00	1.00	1.00	1.00	1.00	1.00	1.00	1.00	1.00	1.00	1.01
Familial diabetes (yes; ref: no)	0.71	0.36	1.40	1.11	0.79	1.55	0.73	0.49	1.09	0.71	0.25	1.99
Familial dyslipidemia (yes; ref: no)	0.96	0.63	1.46	**1.24**	**1.01**	**1.52**	0.98	0.77	1.26	1.08	0.57	2.03
Familial hypertension (yes; ref: no)	**2.13**	**1.45**	**3.12**	**1.26**	**1.05**	**1.52**	**2.08**	**1.67**	**2.60**	**3.33**	**1.87**	**5.91**
Fruit/vegetable consumption	1.01	0.98	1.03	1.01	0.99	1.04	1.01	0.99	1.04	1.02	0.99	1.05
Age × fruit/vegetable consumption	0.99	0.99	1.00	0.99	0.99	1.00	0.99	0.99	1.00	0.99	0.98	1.00
Processed food consumption	0.98	0.94	1.03	1.01	0.96	1.06	0.98	0.93	1.03	0.99	0.93	1.04
Number of media (≥1; ref: 0)	1.09	1.00	1.19	0.98	0.90	1.08	1.05	0.96	1.16	**1.30**	**1.18**	**1.43**
Number of media × age	**1.09**	**1.05**	**1.13**	**1.05**	**1.02**	**1.09**	0.99	0.95	1.02	**1.18**	**1.14**	**1.23**
Well-being score	**0.90**	**0.82**	**0.98**	1.01	0.92	1.10	0.91	0.83	1.00	0.91	0.82	1.02
Well-being score × age	0.99	0.97	1.02	0.99	0.96	1.01	1.01	0.98	1.04	0.97	0.95	1.00
Sports club (no; ref: yes)	1.08	0.99	1.17	**1.16**	**1.07**	**1.26**	1.08	0.99	1.18	**1.30**	**1.18**	**1.42**
Sports club × age	0.99	0.96	1.01	1.02	0.99	1.04	1.01	0.98	1.03	0.97	0.94	0.99
CRP *z*-score	**1.40**	**1.31**	**1.49**	**1.16**	**1.10**	**1.22**	**1.18**	**1.12**	**1.24**	**1.59**	**1.49**	**1.70**
CRP z-score × age	**0.97**	**0.95**	**0.99**	1.01	0.99	1.02	**0.96**	**0.95**	**0.98**	**0.93**	**0.91**	**0.96**

Continuous variables were centered (and rescaled) to: 8 years of age, 3500 g birth weight (1 unit~ 100 g), 6 months of breastfeeding, maternal BMI of 23 kg/m^2^, eating processed food 0 times/day, eating fruits and vegetables 5 times/day, well-being score of 40 (1 unit~10 points).

Statistically significant results are shown in bold (95% confidence interval does not include the value 1).

Models are mutually adjusted except for CRP *z*-score; CRP *z*-score was added only in a second step such that effect estimates show the direct effects of the lifestyle factors not mediated through CRP *z*-score but the effect of CRP *z*-score is adjusted for lifestyle factors. All models were additionally adjusted for sex and country of origin. Estimates were corrected for multiple imputation.

*CRP* C-reactive protein, *ISCED* International Standard Classification of Education, *LCL* lower 95% confidence limit, *MetS* metabolic syndrome, *OR* odds ratio, *ref* reference category, *UCL* upper 95% confidence limit.

Among the lifestyle factors, having at least one media in the bedroom markedly increased the risk of showing several MetS components; the OR strongly increased with age (1.30 [1.18; 1.43] at age 8; 1.18 [1.14; 1.23] for interaction with age; i.e., OR of 1.30 × 1.18 = 1.53 at age 9,…, 1.30 × 1.18^5^ = 2.97 at age 13). Not being member in a sports club increased the risk for dyslipidemia (1.16 [1.07; 1.26]) as well as for showing several MetS components (1.30 [1.18; 1.42]) at age 8. In contrast, a better well-being was associated with a reduced risk of having abdominal obesity (0.90 [0.82; 0.98]). The majority of associations observed did not markedly change with age; a strong age-dependency was only revealed for bedroom media as well as puberty entry. No associations were found for breastfeeding duration, fruit and vegetable or processed food consumption with any of the metabolic outcomes of interest in the mutually adjusted models.

## Discussion

Our data of the large European IDEFICS/I.Family cohort with comprehensive phenotyping and clinical metabolic blood markers even in young children provided the unique opportunity to estimate the age-dependent associations between multiple risk factors and four distinct metabolic health statuses in the transition phase from childhood to adolescence.

Our results confirm that both lifestyle factors, in particular PA, SB and well-being, as well as non-modifiable risk factors, i.e., maternal BMI, parental education, pubertal status, and familial hypertension, are particularly relevant for the development of metabolic disturbances in children and adolescents. Interestingly, the strongest associations between lifestyle factors and metabolic outcomes were observed for the proxy exposures of PA and SB, i.e., being a member of a sports club and bedroom media. This is in line with previous results indicating that PA is inversely and SB positively associated with elevated metabolic risk [4, 5, 40]. Having at least one media in the bedroom markedly increased the risk for abdominal obesity and for showing several MetS components. While most of the associations observed did not markedly change with age, here the OR was more than doubled comparing 8-year-old versus 13-year-old children. This may reflect a generally higher media use with increasing age and its resulting adverse effects (e.g., increased snacking, higher SB, lower PA, reduced sleep duration, and subsequent weight gain) on metabolic health outcomes [41–43]. The other variable showing a large age-dependency was puberty entry. In line with previous studies [44, 45], entering puberty at an early age additionally increased the risk for metabolic disturbances. However, this may also indicate reverse causality, as adiposity is positively associated with sexual maturation, at least in girls [46, 47].

Additionally, confirming previous studies [48–50], a higher well-being score was found to reduce the risk for abdominal obesity. This association may be explained by a more favorable diet, less sedentary time and increased PA in children with higher well-being [51] as low well-being, and particularly stress, lead to unhealthier lifestyle behaviors [52].

Blood lipid dysregulations were mainly influenced by puberty entry, maternal BMI and familial dyslipidemia. Out of the lifestyle factors, only not being member of a sports club was found to be associated with dyslipidemia. This is in line with previous studies [53] reporting that high PA reduces the risk of dyslipidemia.

Interestingly, reducing the risk of hypertension may be difficult as none of the considered lifestyle factors but only CRP, maternal BMI and familial predisposition were found to be influencing factors. However, salt intake is known to affect hypertension and was not (directly) considered here, i.e., only indirectly using the proxy measure of processed food [54]. It should be further noted that the hypertensive group considered here consists of children showing a high probability of hypertension but not of obesity. In these children hypertension may indeed be mainly genetically driven (whereas hypertension as a co-morbid condition of obesity may be strongly related to lifestyle).

In summary, our findings support the promising potential for public health efforts in children and adolescents focusing specifically on increasing PA, decreasing SB and on the strengthening of psychological well-being. Additionally, low parental education and high maternal BMI showed strong positive associations with children’s metabolic risk. Maternal BMI may be a proxy for the familial environment, but may also partly reflect genetic factors. Both education as well as maternal weight status can possibly be improved through political measures. For instance, enabling access to higher education and health promotion programs to socially disadvantaged future parents may reduce the children’s risk of overweight/obesity [55]. Additionally, situational prevention promoting healthy lifestyles, e.g., in terms of taxing sugar-sweetened beverages [56] and fast food, as well as lowering of fruit and vegetable prices [57] has been shown to beneficially influence dietary behaviors.

The strong associations we observed between metabolic disturbances and CRP *z*-scores are in line with other studies, which have shown that CRP levels in children and adolescents are positively associated with abdominal obesity, dyslipidemia [58] and other MetS risk factors [59–61]. We included CRP as an exposure variable as high levels are assumed to promote the incidence of MetS components [13, 62] and of obesity, as previously shown in IDEFICS children [15]. However, to some extent there may be reverse causality. Abdominal obesity can also lead to elevated CRP as a result of macrophage infiltration in adipose tissue that release inflammatory signals and cytokines [63], which has been shown in children with obesity from 6 years onwards [64].

### Limitations and strengths

Our study is not free of limitations. To begin with, to avoid increasing model complexity, only linear age-dependent relationships were modeled. In addition, the probabilities estimated in the LTA (stage 1) were used in the multivariate mixed model (stage 2) without accounting for the uncertainty of model estimates in stage 1. Further, puberty entry, an important non-modifiable risk factor, was only assessed at T3 from the age of 8 years onwards. Additionally, all lifestyle factors, early life factors, and parental characteristics were proxy-/self-reported, thus reporting errors and social desirability cannot be precluded. Our sample included few children with a low educational level of parents leading to instable estimates. This was the reason to combine low and medium educational levels into one category.

In the present analysis, children were defined as being above the so-called “monitoring” or “action” levels of the different metabolic parameters if the parameters exceeded the 90th or 95th age- and sex-specific reference percentiles that were derived based on data from healthy IDEFICS/I.Family children [1]. As we applied this definition to a subset of the IDEFICS/I.Family cohort in the present study, it is expected that at least 10% of the children fall above the monitoring level and 5% above the action level.

An important strength of our unique study is the large sample of young children and adolescents with comprehensive longitudinal data on lifestyle, early life factors, parental variables and clinical biomarkers. This allowed us to assess the contribution of various factors to the risk of showing four distinct metabolic disturbances during the transition from childhood to adolescence. Applying LTA, a sophisticated statistical approach, helped to reduce the dimensionality of data with respect to the 16 possible combinations of presence and absence of the considered metabolic risk factors, and to derive easily interpretable distinct metabolic statuses. To our knowledge this is the first study combining LTA with a compositional data approach. The use of (transformed) probabilities of assignment to the different latent groups as outcome variables in a multivariate model has the great advantage that it accounts for the uncertainty of the latent group assignment but still results in OR as measures of association.

## Conclusion

Using innovative statistical models, our study demonstrated the age-dependent contribution of multiple lifestyle and non-modifiable risk factors as well as CRP to the risk for metabolic disturbances in a large sample of 4- to 15-year-old children. Children’s media in bedroom, well-being as well as membership in a sports club appeared to be the most relevant lifestyle factors, while maternal BMI, family history of hypertension, parental education, and puberty entry were the most relevant non-modifiable factors. Our results demonstrate the need for multifactorial interventions. In this regard, in particular high-risk groups such as children from families with low socioeconomic status or with maternal obesity may benefit most from public health and education policies.

## Supplementary information

Supplementary material

## Footnotes

1 Non-modifiable from the child’s perspective.

2 Non-modifiable from the child’s perspective.

3 In T2, only a postal questionnaire-based survey was conducted such that only data of the examination waves T0, T1, and T3 are considered here.
