# Supplementary material for: The role of lifestyle and non-modifiable risk factors in the development of metabolic disturbances from childhood to adolescence
Source: Int J Obes (Lond). 2020 Sep 17;44(11):2236–45. doi: 10.1038/s41366-020-00671-8 (PMC7577850; doi:10.1038/s41366-020-00671-8)
Supplement: Supplementary file 1 — Supplementary material [file 41366_2020_671_MOESM1_ESM.docx]

**Online Resources**

Article: Börnhorst C, Russo P, Veidebaum T, Tornaritis M, Molnár D, Lissner L, Marild S, De Henauw S, Moreno LA, Floegel A, Ahrens W, Wolters M. The role of lifestyle and non-modifiable risk factors in the development of metabolic disturbances from childhood to adolescence. International Journal of Obesity

Correspondence: Dr. Maike Wolters, Leibniz Institute for Prevention Research and Epidemiology– BIPS, Bremen, Germany; email: wolters@leibniz-bips.de

**Supplementary Material S1: Measurement of outcomes**

*Anthropometric measurements*

Waist circumference [cm] was measured in upright position with relaxed abdomen and feet together, midway between the lowest rib margin and the iliac crest to the nearest 0.1 cm (non-elastic tape: Seca 200; seca, Birmingham, UK).

*Blood pressure*

Blood pressure [mmHg] was measured with an automated oscillometric device (Welch Allyn 4200B-E2, Welch Allyn Inc. NY, USA) where the cuff length was chosen depending on the child’s arm circumference. After at least 5 minutes of resting in a sitting position, two measurements were taken with two minutes interval in between, plus a third one in case the first and second measurements differed by >5%. The average of the two measurements showing the smallest difference was used in the subsequent analysis.

*Collection of blood markers*

Venous blood was collected in a fasting state from children/adolescents. At T0, fasting capillary blood was collected in case children themselves or their parents refused venipuncture.

At T0 and T1, blood glucose, high-density lipoprotein (HDL) and triglycerides were assessed using a point-of-care analyser (Cholestech LDX, Cholestech Corp., Hayward, CA, USA). At T3, an enzymatic UV test (Cobas c701, Roche Diagnostics GmbH, Mannheim, Germany) was used for blood glucose, a homogeneous enzymatic colorimetric test (Cobas c701, Roche Diagnostics GmbH, Mannheim) for HDL and an enzymatic colorimetric test (Cobas c701, Roche Diagnostics GmbH, Mannheim, Germany) for triglycerides. Validation measurements were conducted confirming that the differing laboratory methods used gave similar results. Blood samples were analyzed centrally in a laboratory accredited according to DIN EN ISO 15189 by the German Accreditation Council (at T3).

**Supplementary Material S2: Assessment of determinants of metabolic status**

If not otherwise stated, variables are obtained at T0, T1, and T3 based on proxy-reported questionnaires (in T3 children ≥12 years reported for themselves).

***Lifestyle factors***

Dietary variables

A variable indicating daily consumption frequencies of fruits and vegetables was calculated summing the reported frequencies of a) fresh fruits without sugar added, b) fresh fruits with sugar added, c) cooked vegetables, d) legumes, and e) raw vegetables. Analogously a variable indicating daily consumption frequencies of processed foods was calculated as the sum of a) cold cuts and preserved, meat products, b) snacks like savory pastries and fritters, and c) hamburger, hot dog, kebab, wrap, falafel.

Sports club member

A variable indicating whether the child was member in a sports club was used as proxy for physical activity.

Well-being score

Psychosocial well-being was measured with 16 items of four subscales of the “KINDL-R Questionnaire for Measuring Health-Related Quality of Life (HRQoL) in Children and Adolescents” (emotional well-being, self-esteem, family life and relations to friends).^1,2^ At T3, response categories corresponded to the original 5-point Likert scale (never, seldom, sometimes, often, all the time). At T0 and T1 the two highest response categories were combined into one category. Therefore, we deviated from the original scoring (1-5 points per item) and assigned 0 points for “Never” and 3 points for both “Often” and “All the time” (at follow-up) or “Often/All the time” (at baseline), respectively (six negatively worded items were coded reversely). Consequently, the score ranged from 0-48 with a higher score indicating a higher well-being.

Media

The number of media devices reported to be in children’s’/teens’ bedroom was used as an indicator for media use.^3^ In order to reduce model complexity, a dummy was used in the multivariate model indicating children with 0 vs ≥ 1 media in the bedroom.

***Non-modifiable risk factors***

Demographics

Age of the children, sex, country of residence as well as the highest educational level of parents according to the International Standard Classification of Education (ISCED)^4^ as indicator for socio-economic status were considered. Educational level was used as dummy variable in the multivariate model in order to reduce model complexity (low/medium vs high ISCED level; original coding: 1=low, 2=medium, 3=high).

Family history of diseases

Parents reported the presence of metabolic disturbances for themselves, their partner as well as children. Three variables were constructed indicating the presence of hypertension, dyslipidemia and type 2 diabetes in any of the family members (yes vs. no).

Early life factors

Birth weight (g) and total breast feeding duration (months; including breast feeding combinations) were reported by mothers.

Maternal body mass index (BMI)

Maternal BMI at baseline was calculated as weight (kg) divided by height (m) squared where weight/height were self-reported.

Pubertal status

Only at T3, pubertal status (yes vs. no; yes if menarche had already occurred in girls or if voice alterations had already started or were completed in boys) was self-reported by children 8 years and older based on questions adapted from Carskadon and Acebo.^5^

**Supplementary Material S3**

For missing covariates, multiple imputation was applied using Markov Chain Monte Carlo simulation (MCMC) with 10 replicates. All outcomes and exposures used in the final analyses were included in the multiple imputation procedure. In addition, the BMI z-score according to Cole and Lobstein^6^ was included as an auxiliary variable. The percentages of missing values ranged from 0% (age, sex) up to 37.0% (CRP) depending on the variable considered (see Supplementary Table S4). The relative efficiency was >96.6% for all variables indicating good imputation quality.

**Supplementary Material S4: Description of the study population: Means (SD) for continuous variables and numbers and percentages for categorical variables in the different latent metabolic groups (dataset including missing values)**

| **Status at T0** | **All** |  |  |  | **Metabolically healthy** | | | **Abdominal obesity** | | | **Dyslipidemia** | | | **Hypertension** | | | **Several MetS components** | | |
| --- | --- | --- | --- | --- | --- | --- | --- | --- | --- | --- | --- | --- | --- | --- | --- | --- | --- | --- | --- |
| **Covariate at T0** | **N** | **Missings** | **Mean** | **SD** | **N** | **Mean** | **SD** | **N** | **Mean** | **SD** | **N** | **Mean** | **SD** | **N** | **Mean** | **SD** | **N** | **Mean** | **SD** |
| Maternal BMI | 3670 | 219 | 23.8 | 4.3 | 2501 | 23.3 | 3.9 | 555 | 25.3 | 4.9 | 234 | 23.9 | 4.6 | 182 | 23.6 | 3.8 | 198 | 26.1 | 4.8 |
| Max ISCED of both parents | 3749 | 140 | 2.5 | 0.6 | 2554 | 2.5 | 0.6 | 568 | 2.3 | 0.7 | 241 | 2.5 | 0.6 | 184 | 2.5 | 0.6 | 202 | 2.2 | 0.6 |
| Breast feeding [months] | 3330 | 559 | 5.4 | 6.7 | 2281 | 5.6 | 6.7 | 507 | 4.4 | 6.6 | 202 | 5.1 | 7.1 | 165 | 7.1 | 7.8 | 175 | 3.7 | 5.6 |
| Birth weight (g) | 3679 | 210 | 3343 | 565 | 2514 | 3340 | 558 | 554 | 3369 | 594 | 235 | 3347 | 549 | 176 | 3320 | 575 | 200 | 3317 | 583 |
| Fruit /veg [times/day] | 3186 | 703 | 2.7 | 1.8 | 2175 | 2.7 | 1.7 | 481 | 2.6 | 1.7 | 208 | 2.9 | 2.1 | 159 | 2.8 | 2.1 | 163 | 2.6 | 1.7 |
| Preserved food [times/day] | 3409 | 480 | 1.0 | 0.8 | 2334 | 0.9 | 0.8 | 507 | 0.9 | 0.8 | 217 | 1.0 | 0.7 | 174 | 1.2 | 1.0 | 177 | 1.1 | 1.0 |
| Media in bedroom (N) | 3582 | 307 | 0.9 | 1.2 | 2461 | 0.8 | 1.2 | 534 | 1.2 | 1.3 | 227 | 0.9 | 1.3 | 179 | 0.9 | 1.3 | 181 | 1.2 | 1.4 |
| Well-being score | 3406 | 483 | 39.9 | 4.7 | 2342 | 40.2 | 4.5 | 506 | 39.2 | 4.9 | 212 | 40.0 | 5.0 | 169 | 39.4 | 4.8 | 177 | 39.0 | 4.7 |
| CRP (high-sensitive) [mg/dl] | 2450 | 1439 | 0.14 | 0.39 | 1721 | 0.11 | 0.29 | 337 | 0.25 | 0.7 | 148 | 0.19 | 0.44 | 136 | 0.1 | 0.21 | 108 | 0.24 | 0.39 |
| CRP z-score | 2450 | 1439 | 0.35 | 0.84 | 1721 | 0.23 | 0.78 | 337 | 0.71 | 0.91 | 148 | 0.46 | 0.93 | 136 | 0.26 | 0.75 | 108 | 1.01 | 0.82 |
| **Covariate at T0** | **N** |  | **%** |  | **N** | **%** |  | **N** | **%** |  | **N** | **%** |  | **N** | **%** |  | **N** | **%** |  |
| Missing | 327 |  | 8.4 |  | 184 | 7.0 |  | 57 | 9.7 |  | 33 | 12.8 |  | 18 | 9.4 |  | 35 | 16.2 |  |
| Member in sports club | 1825 |  | 46.9 |  | 1261 | 47.9 |  | 270 | 45.8 |  | 111 | 43.2 |  | 90 | 46.9 |  | 93 | 43.1 |  |
| Not member in sports club | 1737 |  | 44.7 |  | 1190 | 45.2 |  | 262 | 44.5 |  | 113 | 44.0 |  | 84 | 43.8 |  | 88 | 40.7 |  |
| No familial hypertension | 3096 |  | 79.6 |  | 2163 | 82.1 |  | 443 | 75.2 |  | 209 | 81.3 |  | 139 | 72.4 |  | 142 | 65.7 |  |
| Familial hypertension^a^ | 793 |  | 20.4 |  | 472 | 17.9 |  | 146 | 24.8 |  | 48 | 18.7 |  | 53 | 27.6 |  | 74 | 34.3 |  |
| No familial diabetes | 3694 |  | 95.0 |  | 2520 | 95.6 |  | 558 | 94.7 |  | 238 | 92.6 |  | 179 | 93.2 |  | 199 | 92.1 |  |
| Familial diabetes^a^ | 195 |  | 5.0 |  | 115 | 4.4 |  | 31 | 5.3 |  | 19 | 7.4 |  | 13 | 6.8 |  | 17 | 7.9 |  |
| No familial dyslipidemia | 3288 |  | 84.6 |  | 2261 | 85.8 |  | 494 | 83.9 |  | 207 | 80.5 |  | 157 | 81.8 |  | 169 | 78.2 |  |
| Familial dyslipidemia^a^ | 601 |  | 15.5 |  | 374 | 14.2 |  | 95 | 16.1 |  | 50 | 19.5 |  | 35 | 18.2 |  | 47 | 21.8 |  |

^a^No missing values were present for the familial history of hypertension, dyslipidemia and diabetes as parents could only state the presence (but not non-presence) of disease, i.e. not answering the question was considered as non-diseased.

**Supplementary Material S5: Sensitivity analysis**

Our models were in general not adjusted for BMI (weight status) as it is part of the outcome variables (high correlation with abdominal obesity) but also as it lies on the causal pathway between our lifestyle exposures and metabolic outcomes, and mediators are typically not adjusted for.^7^ For instance, diet is known to influence the BMI and BMI is associated with hypertension. However, as BMI may not only be considered a mediator but also a confounder in the association between CRP and hypertension/dyslipidemia, in a sensitivity analysis we estimated separate models for hypertension as well as dyslipidemia with additional adjustment for BMI z-score and its age interaction. Results were not altered by this additional adjustment. This was expected as according to the latent transition model, children in the hypertensive/dyslipidemia groups were mainly normal weight making weight status unlikely to alter the observed associations.

**References**

1. Bullinger M, Brutt AL, Erhart M, Ravens-Sieberer U, Group BS. Psychometric properties of the KINDL-R questionnaire: results of the BELLA study. *Eur Child Adolesc Psychiatry* 2008; **17 Suppl 1:** 125-32.

2. Ravens-Sieberer U, Bullinger M. Kindl-R English questionnaire for measuring health-related quality of life in children and adolescents. Revised Version Manual. . In: Ulrike Ravens-Sieberer & Monika Bullinger, 2000.

3. Santaliestra-Pasias AM, Mouratidou T, Verbestel V, Bammann K, Molnar D, Sieri S *et al.* Physical activity and sedentary behaviour in European children: the IDEFICS study. *Public Health Nutr* 2014; **17**(10)**:** 2295-306.

4. United Nations Educational Scientific and Cultural Organization (UNESCO). International Standard Classification of Education, ISCED 2011. In. Canada: UNESCO Institute for Statistics, 2012.

5. Carskadon MA, Acebo C. A self-administered rating scale for pubertal development. *J Adolesc Health* 1993; **14**(3)**:** 190-5.

6. Cole TJ, Lobstein T. Extended international (IOTF) body mass index cut-offs for thinness, overweight and obesity. *Pediatr Obes* 2012; **7**(4)**:** 284-94.

7. Westreich D, Greenland S. The table 2 fallacy: presenting and interpreting confounder and modifier coefficients. *Am J Epidemiol* 2013; **177**(4)**:** 292-8.
